# Impact of long-term androgen deprivation therapy on PSMA ligand PET/CT in patients with castration-sensitive prostate cancer

**DOI:** 10.1007/s00259-018-4079-z

**Published:** 2018-07-07

**Authors:** Ali Afshar-Oromieh, Nils Debus, Monika Uhrig, Thomas A. Hope, Michael J. Evans, Tim Holland-Letz, Frederik L. Giesel, Klaus Kopka, Boris Hadaschik, Clemens Kratochwil, Uwe Haberkorn

**Affiliations:** 1 Department of Nuclear Medicine, Heidelberg University Hospital, Heidelberg, Germany; 2 Department of Nuclear Medicine, Bern University Hospital, Freiburgstrasse 18, 3010 Bern, Switzerland; 3 Department of Radiology, German Cancer Research Center, Heidelberg, Germany; 4 Department of Radiology and Biomedical Imaging, University of California, San Francisco, San Francisco, California USA; 5 Department of Pharmaceutical Chemistry, University of California, San Francisco, San Francisco, California USA; 6 Department of Biostatistics, German Cancer Research Center, Heidelberg, Germany; 7 Division of Radiopharmaceutical Chemistry, German Cancer Research Center, Heidelberg, Germany; 8 German Cancer Consortium (DKTK), Heidelberg, Germany; 9 Department of Urology, Essen University Hospital, University of Duisburg-Essen, Essen, Germany; 10 Clinical Cooperation Unit Nuclear Medicine, German Cancer Research Centre, Heidelberg, Germany

**Keywords:** Prostate cancer, PET/CT, PSMA, Prostate-specific membrane antigen, ^68^Ga-PSMA-11, Androgen deprivation therapy

## Abstract

**Purpose:**

Since the introduction of PSMA PET/CT with ^68^Ga-PSMA-11, this modality for imaging prostate cancer (PC) has spread worldwide. Preclinical studies have demonstrated that short-term androgen deprivation therapy (ADT) can significantly increase PSMA expression on PC cells. Additionally, retrospective clinical data in large patient cohorts suggest a positive association between ongoing ADT and a pathological PSMA PET/CT scan. The present evaluation was conducted to further analyse the influence of long-term ADT on PSMA PET/CT findings.

**Methods:**

A retrospective analysis was performed of all 1,704 patients who underwent a ^68^Ga-PSMA-11 PET/CT scan at our institution from 2011 to 2017 to detect PC. Of 306 patients scanned at least twice, 10 had started and continued ADT with a continuous clinical response between the two PSMA PET/CT scans. These ten patients were included in the current analysis which compared the tracer uptake intensity and volume of PC lesions on PSMA PET/CT before and during ongoing ADT.

**Results:**

Overall, 31 PC lesions were visible in all ten patients before initiation of ADT. However, during ongoing ADT (duration 42–369 days, median 230 days), only 14 lesions were visible in eight of the ten patients. The average tracer uptake values decreased in 71% and increased in 12.9% of the PC lesions. Of all lesions, 33.3% were still visible in six patients with a complete PSA response (≤0.1 ng/ml).

**Conclusion:**

Continuous long-term ADT significantly reduces the visibility of castration-sensitive PC on PSMA PET/CT. If the objective is visualization of the maximum possible extent of disease, we recommend referring patients for PSMA PET/CT before starting ADT.

## Introduction

The detection of recurrent prostate cancer (PC) has always been challenging using conventional imaging modalities such as computed tomography (CT) and magnetic resonance imaging. Consequently, there has been a need for new imaging tools with improved sensitivity and specificity in diagnosis. In this context, nuclear imaging targeting prostate-specific membrane antigen (PSMA) has received increased attention. PSMA is a transmembrane protein which is overexpressed in the majority of aggressive prostatic adenocarcinomas [1–3]. In May 2011, the PSMA ligand ^68^Ga-PSMA-11 was introduced for the clinical imaging of PC at our institution. Since then, positron emission tomography (PET) using ^68^Ga-PSMA-11 and alternative PSMA ligands has been regarded as a significant step forward in the diagnosis of recurrent PC. The first studies indicated that this novel method is superior to alternative imaging modalities used for the detection of recurrent PC [4–6]. Later studies including larger patient cohorts confirmed the high sensitivity and specificity of ^68^Ga-PSMA-11 PET/CT [7–10].

One of the most important questions in PSMA imaging concerns the role of androgen deprivation therapy (ADT). It is known from preclinical experiments that short-term ADT can quantitatively increase PSMA expression in castration-sensitive PC cells [11, 12]. This effect was shown with analogues of the luteinizing hormone-releasing hormone (LHRH) as well as with antiandrogen agents such as bicalutamide and enzalutamide. In addition, Hope et al. demonstrated in one patient, that PSMA ligand uptake in PC lesions significantly increased within 4 weeks of ADT with bicalutamide [13]. On the other hand, the effects of long-term ADT on PSMA expression and tumour visibility in PSMA ligand PET/CT have not yet been investigated in detail. Two clinical studies including larger patient cohorts (*n* = 317 and 1,007) demonstrated that patients during ongoing ADT significantly more often had a pathological ^68^Ga-PSMA-11 PET/CT scan [6, 7]. The authors considered that this association could have been a result of either a possible biological impact of ADT (increase of PSMA expression similar to the findings of the preclinical experiments with short-term ADT mentioned above) or the fact that patients with a pathological scan had more advanced tumour stages which had led to the initiation of ADT.

The aim of the current evaluation was to directly analyse the clinical impact of long-term ADT on tumour visibility and tumour detection on ^68^Ga-PSMA-11 PET/CT. To the best knowledge of the authors, no systematic studies have been published including patients who were scanned with PSMA ligand PET/CT before and after starting ADT.

## Materials and methods

### Patients and inclusion criteria

**Fig. 1:**
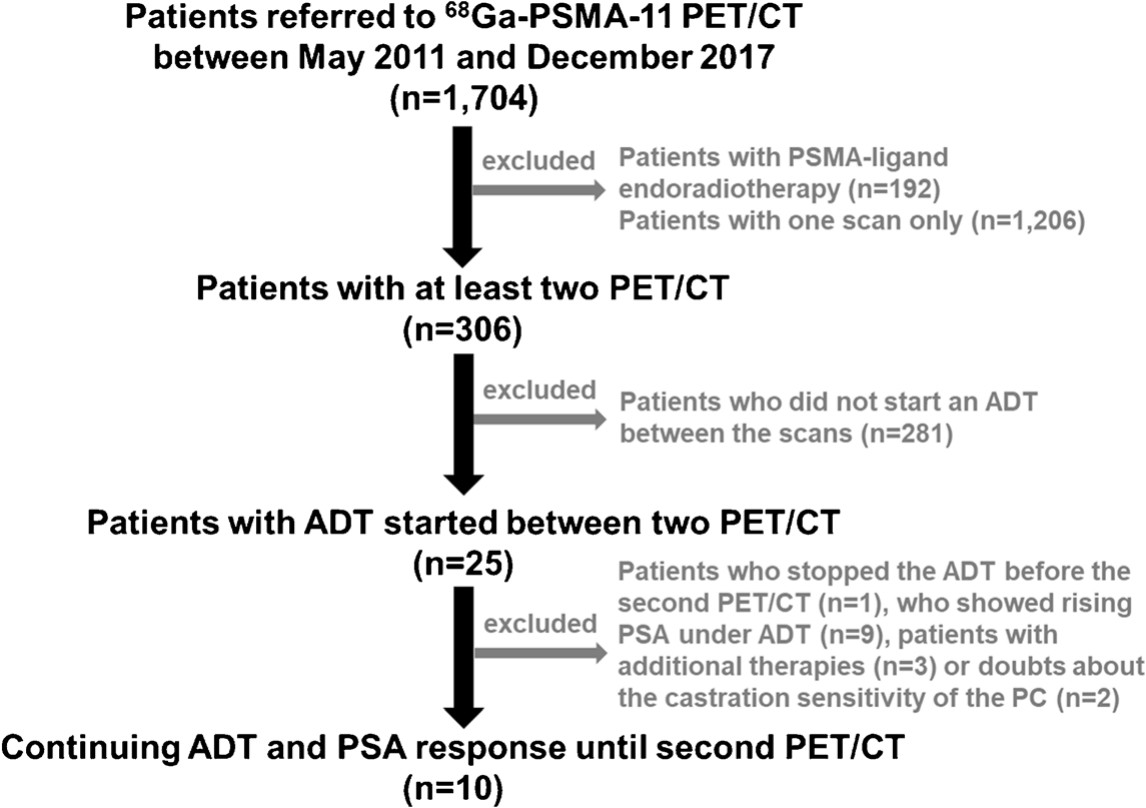
Patient selection flow chart

We retrospectively analysed all 1,704 patients who were scanned with ^68^Ga-PSMA-11 PET/CT at our department from May 2011 to December 2017 to detect PC. Patients who were referred for PSMA radioligand therapy were excluded from the analysis (*n* = 192). Of the remaining patients, 306 were scanned at least twice, and 25 of these had started ADT between the two ^68^Ga-PSMA-11 PET/CT scans. Of these 25 patients, 10 continued ADT and showed a PSA response until the second PET/CT, and were therefore included in the current analysis. The other 15 of the 25 patients were excluded from the analysis due to pausing ADT before the second PET/CT scan, increasing PSA levels indicating castration resistance or other additional therapies including chemotherapy. The process of patient selection for inclusion is shown in Fig. 1. The characteristics of the ten patients including the ADT drugs they received are summarized in Table 1. The patient had previous radiation therapy of the primary PC 3.5 years before the first PET scan. The radiation therapy was accompanied by ADT which was continued until 24 months before the first PET scan. All patients had been partially analysed in previous studies investigating different aspects [4–7, 14, 15].

**Table 1:** Characteristics of the ten included patients

Patient no.	Age (years)	^68^Ga-PSMA-11 (MBq)		Gleason score	PSA (ng/ml)		Time PET-1 to PET-2 (days)	Treatments prior to PET-1	Number of PC lesions		ADT between PET-1 and PET-2	
		PET-1	PET-2		PET-1	PET-2			PET-1	PET-2	Drug	Duration (days)
1	70	119	136	7	13.90	1.20	343	RT	2	1	Bicalutamide and leuprorelin	323
2	73	194	190	9	313.13	14.00	314	None	2	1	Bicalutamide and leuprorelin	278
3	70	341	212	8	10.50	1.90	309	RPx, RT	5	4	Leuprorelin	231
4	68	110	282	6	1.69	<0.1	377	RT + ADT^a^	5	0	Leuprorelin	369
5	68	225	211	9	5.20	0.10	207	RT	1	1	Leuprorelin	177
6	66	313	203	7	13.80	<0.1	270	RT	3	3	Bicalutamide	223
7	64	271	230	9	3.10	0.39	102	RPx	1	1	Bicalutamide	42
8	71	221	259	7	5.60	0.10	196	RPx	6	2	Leuprorelin	184
9	77	195	235	7	3.10	0.10	236	RPx	5	1	Bicalutamide	228
10	57	156	226	8	0.25	0.02	334	RPx, RT	1	0	Triptorelin	235

All patients showed decreasing PSA levels between the first and second PET scan

*RT* Radiation therapy of the prostate or prostate fossa, *RPx* radical prostatectomy

^a^Patient 4 had previous radiation therapy 3.5 years before the first PET scan, accompanied by ADT which was continued until 24 months before the first PET scan

### Radiotracer and imaging

^68^Ga-PSMA-11 was produced and PET/CT scans were performed as previously described [7, 16].

### Analysis of images and PC lesions

In a first round, two board-certified specialists in nuclear medicine with 13 and >20 years of clinical experience (first and last author) read all datasets together and resolved any disagreements by consensus. Lesions that were visually considered as suggestive of PC were counted and analysed with respect to their localization (local relapses, lymph node, bone and soft tissue metastases) as well as to their average and maximum standardized uptake values (SUVmean and SUVmax) which represent the intensity of PSMA ligand accumulation in PC lesions. SUV in late images was categorized as increasing, decreasing or stable with intensity changes of >10%, <−10% or between −10% and +10%, respectively. In a second round, two board-certified specialists in nuclear medicine and radiology (first and third author) read all datasets together and analysed the lesions with respect to their volumes (measured using the ellipsoid method by multiplying the three perpendicular axes A, B, and C and dividing the result by 2). Any disagreements were resolved by consensus. The tumour volumes were also categorized as increasing, decreasing or stable with intensity changes of >10%, <−10% or between −10% and +10%, respectively.

### Statistical analysis

Significance of differences between PC lesions before and after ADT with regard to SUVs (mean and maximum), lesion size, lesion type (lymph nodes or bone metastases; local relapses and soft tissue metastases were excluded due to low numbers), and the SUV to lesion size ratios were evaluated using a linear mixed model including patient ID and lesion ID as random factors (package ‘lme4’ of R version 3.4.0 software). Thus, the possible effects of multiple lesions per patient were accounted for. As most values showed a somewhat skewed distribution, log-transformation was applied to all variables before statistical testing, resulting in approximately normal distributions. For the transformation, the SUVs of lesions which were PET-negative (no uptake above local background) were treated as 0.1. PSA levels and number of lesions were compared between time points using the paired two-sided Wilcoxon signed ranks test. A *p* value of <0.05 was considered statistically significant.

## Results

The average duration of ADT before the second PSMA PET/CT scan was 229 ± 89 days (range 42–369 days, median 230 days). All patients had at least one lesion characteristic of PC on the initial PET/CT scan (without ADT). Overall, 31 PC lesions were visible in ten patients on the PET/CT scan before initiation of ADT. During ongoing and effective ADT, as demonstrated by a decreasing PSA level, 14 of the 31 lesions (45%) were still visible in eight of the ten patients (80%) on the second PET/CT scan (Table 1). The lower number of tumour lesions during ongoing ADT was statistically significant (*p* = 0.0199). Amongst all lesions characteristic of PC, 19 were lymph node metastases (eight still visible during ongoing ADT), ten were bone metastases (five still visible during ongoing ADT), one was a soft tissue metastasis (lung, no longer visible during ongoing ADT), and one was a primary tumour (also still visible during ongoing ADT). Even though the overall number of lesions was low, the statistical analysis showed that lymph node metastases were more likely to become no longer visible than bone metastases (*p* = 0.0499).

**Fig. 2:**
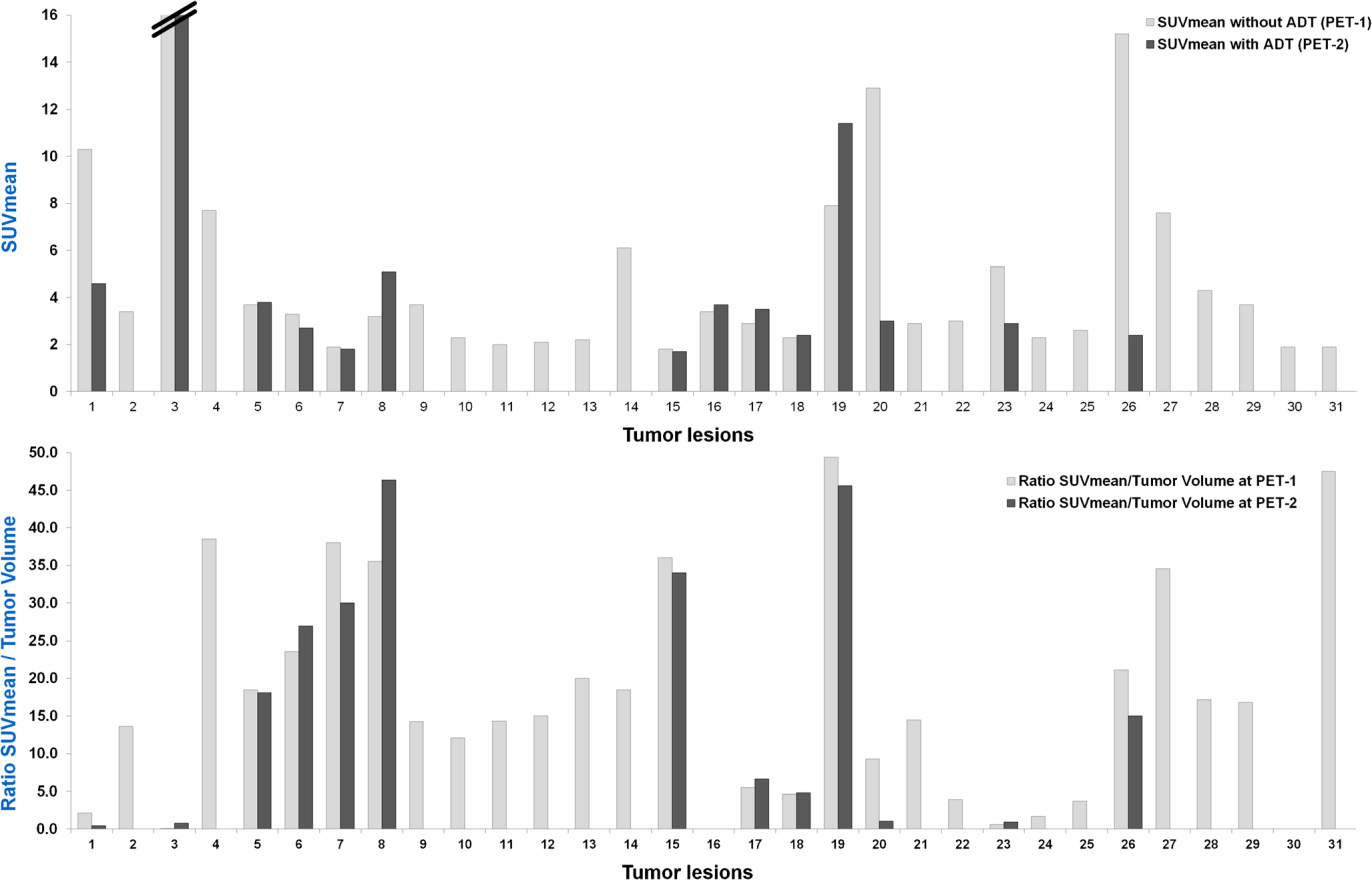
The majority of PC lesions show clearly decreasing average tracer uptake (SUVmean, *top*) and tracer uptake to tumour volume ratios (*bottom*) during ongoing ADT. No bars are shown for those lesions that were no longer visible during ongoing ADT (on PET-2) or for those lesions without a clear morphological correlate in CT (impossible volume measuring). SUVmean values for the cut bar (number 3) were 20.3 on PET-1 and 27.2 on PET-2. *Lesion allocations*: lesions 1 and 2 (patient 1), lesions 3 and 4 (patient 2), lesions 5–9 (patient 3), lesions 10–14 (patient 4), lesion 15 (patient 5), lesions 16–18 (patient 6), lesion 19 (patient 7), lesions 20–25 (patient 8), lesions 26–30 (patient 9) and lesion 31 (patient 10)

**Fig. 3:**
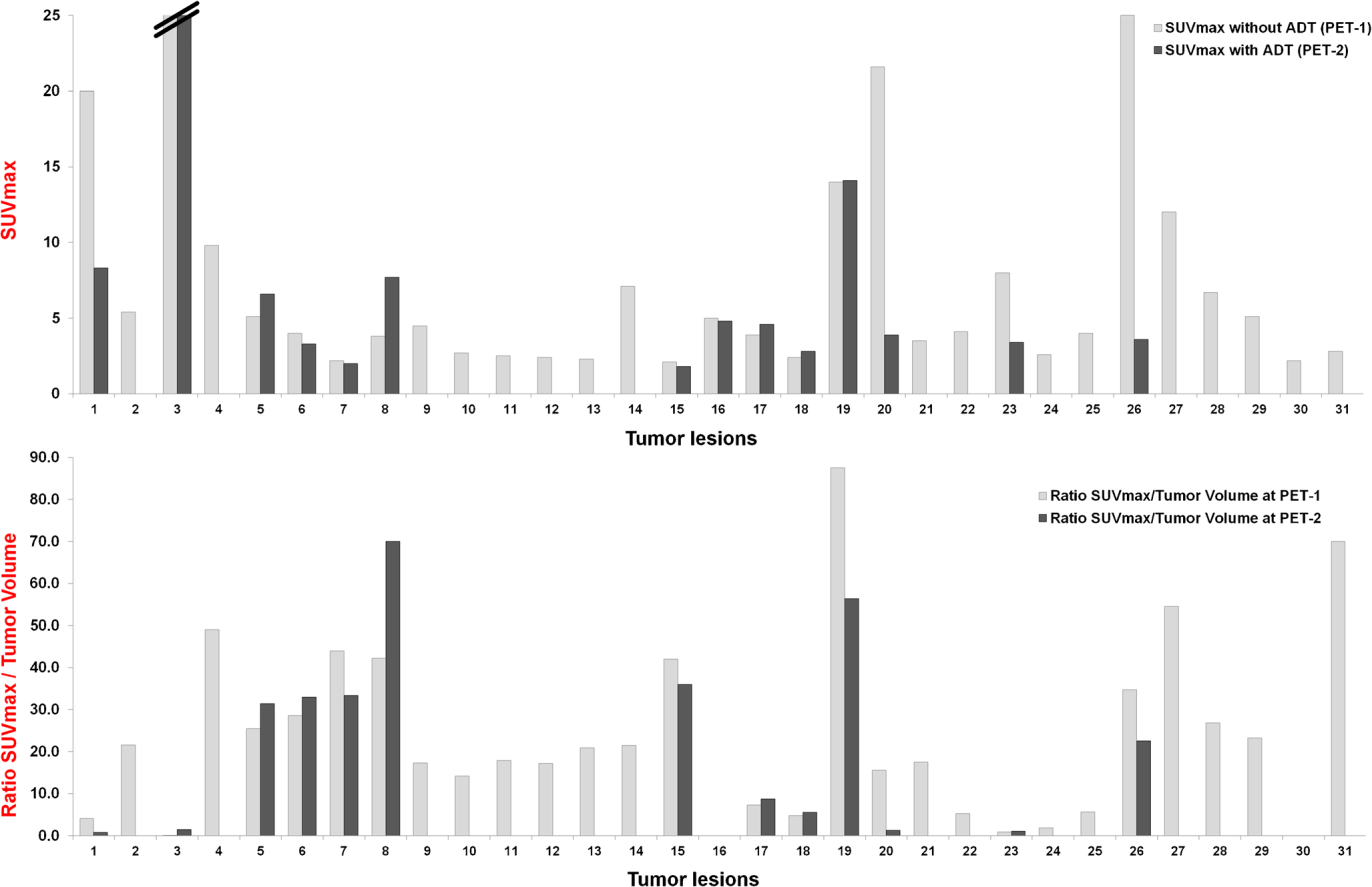
The majority of PC lesions show clearly decreasing maximum tracer uptake (SUVmax, *top*) and tracer uptake to tumour volume ratios (*bottom*) during ongoing ADT. No bars are shown for lesions that were no longer visible during ongoing ADT (on PET-2)

**Fig. 4:**
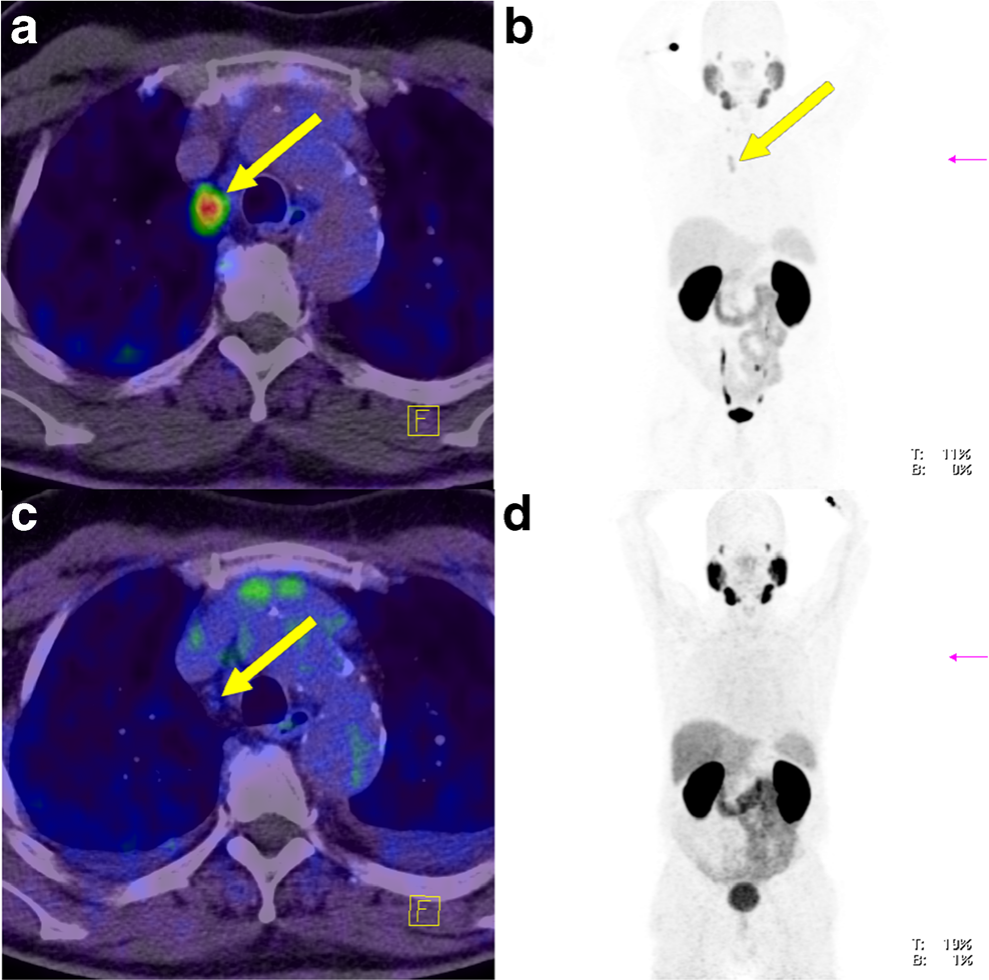
Lymph node metastasis in an example patient (patient 8; *yellow arrows*) without ADT (**a**, **b**) and during ongoing ADT with a complete PSA response (**c**, **d**) 190 days after the first scan. During ongoing ADT, both, tracer uptake intensity and tumour volume significantly decreased. **a** Fused PET and CT image without ADT. **b** Maximum intensity projection image of the PET data without ADT. **c** Fused PET and CT image during ongoing ADT. **d **Maximum intensity projection image of the PET data during ongoing ADT

**Fig. 5:**
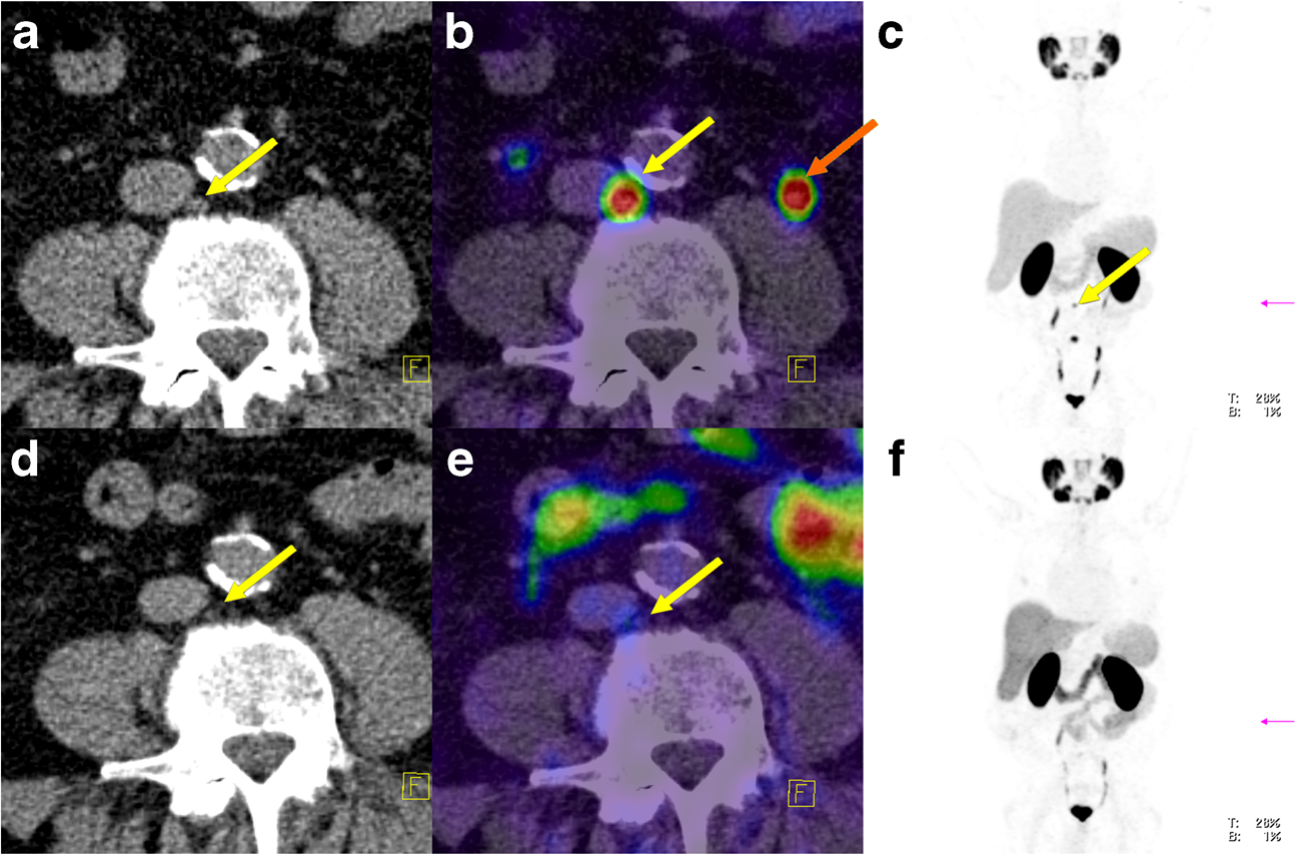
Lymph node metastasis in an example patient (patient 9) without ADT (**a**–**c**) and during ongoing ADT with a complete PSA response (**d**–**f**) 228 days after the first scan (*orange arrow* left ureter). **a** Low-dose CT image without ADT. **b** Fused PET and CT image without ADT. **c** Maximum intensity projection image of the PET data without ADT. **d–f** same as **a–c** but during ongoing ADT

**Fig. 6:**
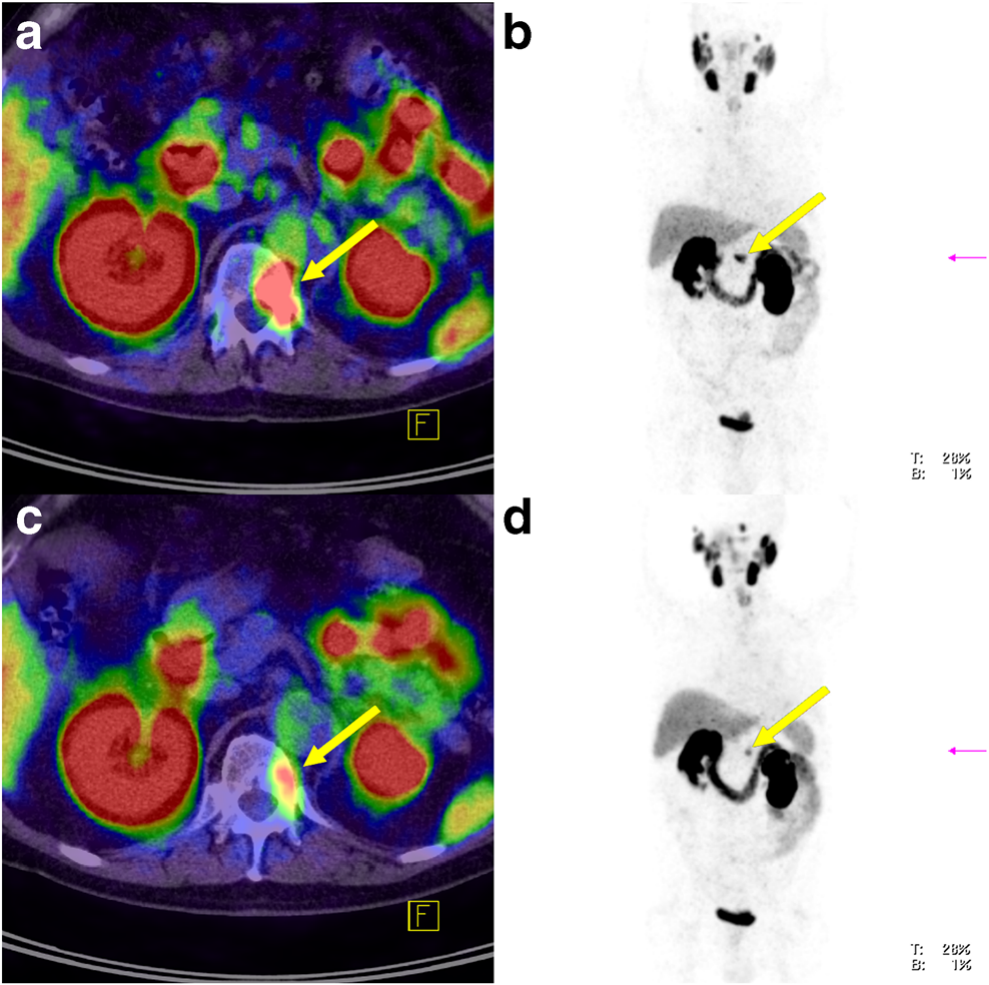
Bone metastasis in an example patient (patient 1; *yellow arrows*) without ADT (**a**, **b**) and during ongoing ADT (**c**, **d**) 323 days after the first scan. **a** Fused PET and CT image without ADT. **b** Maximum intensity projection image of the PET data without ADT. **c** Fused PET and CT image during ongoing ADT. **d** Maximum intensity projection image of the PET data during ongoing ADT

During ongoing ADT, SUVmean increased in four lesions (12.9%), decreased in 22 (71%) and remained stable in five (16.1%; Fig. 2). SUVmax increased in five lesions (16.1%), decreased in 23 (74.2%) and remained stable in three (9.7%; Fig. 3). The decrease in both SUVmean and SUVmax was statistically significant (*p* < 0.0001). Figures 4, 5 and 6 present examples of the negative impact of continuous ADT on tumour visibility on PSMA ligand PET/CT.

Between PET-1 (without ADT) and PET-2 (during ongoing ADT) the SUVmean to tumour volume ratio increased in six lesions (19.4%) and decreased in 23 (74.2%; Fig. 2). In two lesions, the ratio was not calculable because of a complete morphological response to the ADT. The same results were found for the SUVmax to tumour volume ratio (Fig. 3). The decrease in the SUV to tumour volume ratio during ongoing ADT was also statistically significant (*p* = 0.0009 for SUVmean and *p* = 0.0007 for SUVmax).

Between PET-1 (without ADT) and PET-2 (during ongoing ADT) tumour volume increased in ten lesions (32.3%), decreased in 15 (48.4%) and remained unchanged in six (19.3%). The decrease in tumour volume during ongoing ADT was statistically significant (*p* = 0.0015). Of the ten lesions that showed an increase in volume, two showed an increase in SUVmean (one also with an increase in SUVmax), two showed stable SUVmean (one also with stable SUVmax) and six showed decreases in both SUVmean and SUV max. Of the 15 lesions that showed a decrease in volume, one showed increases in both SUVmean and SUVmax, and 14 showed a decrease in SUVmean and SUVmax. Overall, two lesions showed an increase in volume and in tracer uptake, six lesions showed an increase in volume and in tracer uptake and two lesions showed an increase in volume and stable tracer uptake.

**Fig. 7:**
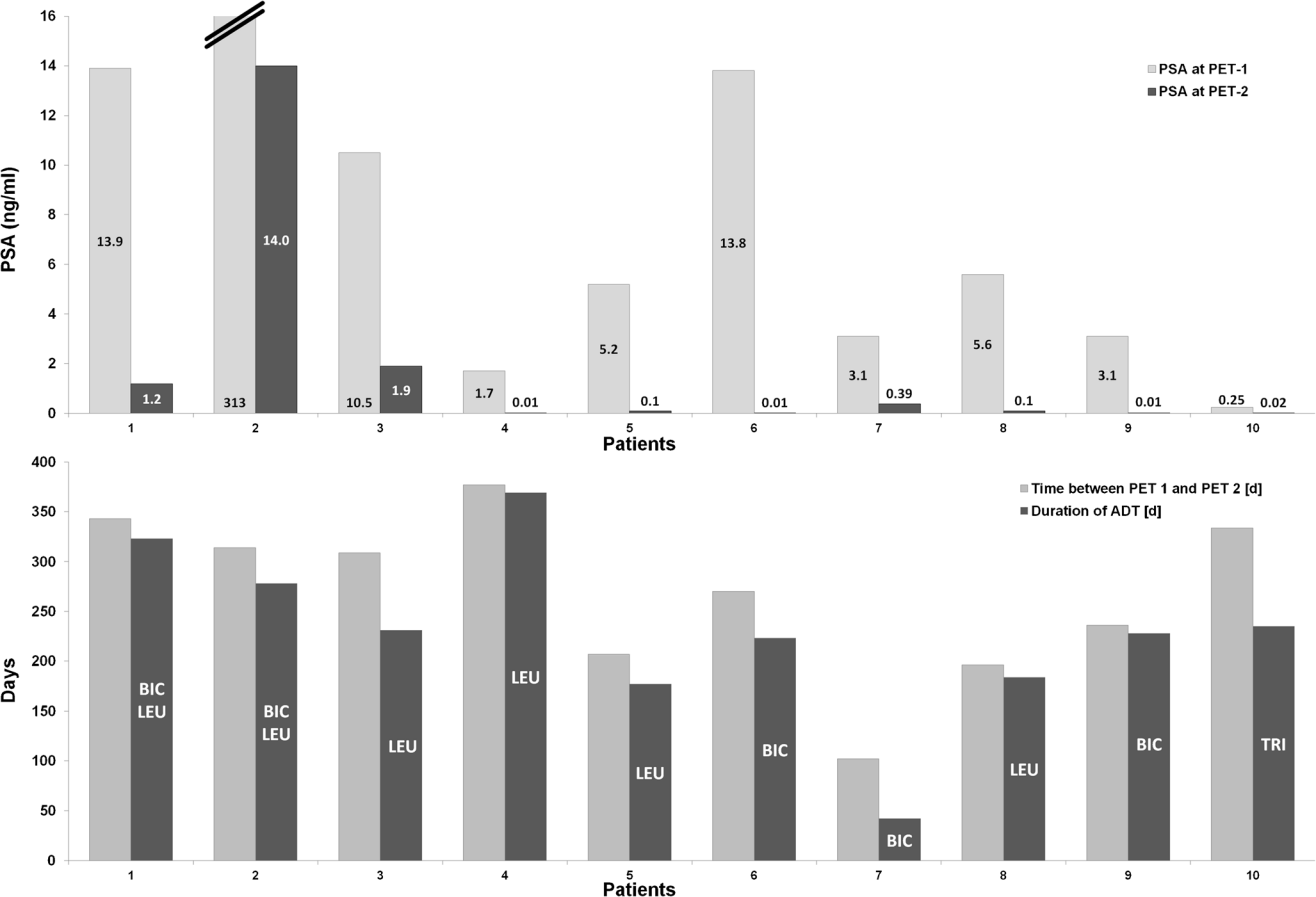
A PSA response to ADT was observed in all patients (*top*). A complete imaging response (negative PSMA PET/CT) was observed in patient 4 and 10 although more patients showed a complete PSA response (*BIC* bicalutamide, *LEU* leuprorelin, *TRI* triptorelin)

**Fig. 8:**
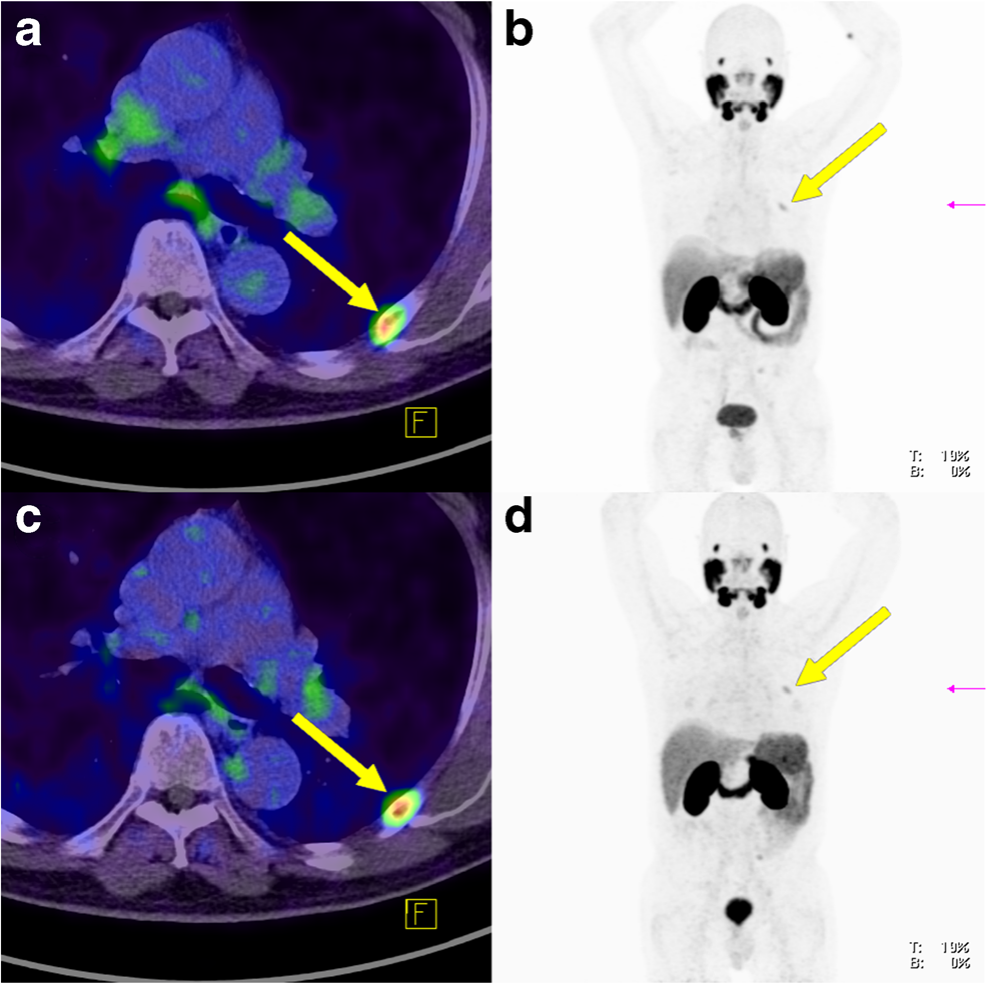
Bone metastasis in an example patient (patient 6; *yellow arrows*; lesion 16 in Fig. 2) visible on both PET-1 and PET-2 with similar tracer uptake despite a complete PSA response (0.01 ng/ml) to ADT (**a**, **b** PET-1 without ADT; **c**, **d** PET-2 during ongoing ADT 270 days after the first scan). **a** Fused PET and CT image without ADT. **b** Low-dose CT image without ADT. **c** Fused PET and CT image during ongoing ADT. **d** Low-dose CT image during ongoing ADT

**Fig. 9:**
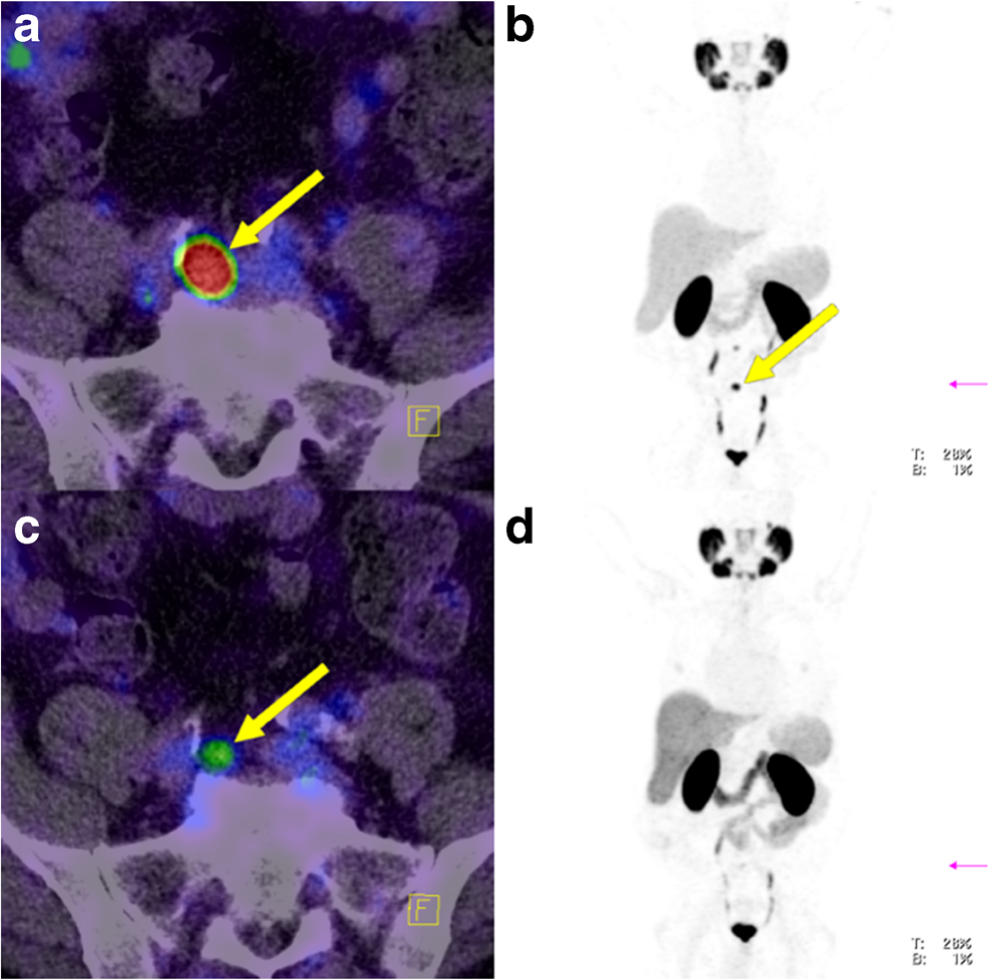
Lymph node metastasis in an example patient (patient 9; *yellow arrows*) clearly visible on PET-1 without ADT (**a**, **b**) which became significantly less visible on PET-2 with a complete PSA response to ADT (**c**, **d**). **a** Fused PET and CT image without ADT. **b** Maximum intensity projection image of the PET data without ADT. **c** Fused PET and CT image during ongoing ADT. **d** Maximum intensity projection image of the PET data during ongoing ADT

PSA levels showed a significant decrease in all patients (*p* = 0.0001; Fig. 7). Considering a PSA level of 0.1 ng/ml or lower as a complete response, six patients, patients 4–6 and 8–10 (Fig. 7) with an ADT duration of 184–369 days (average of 236 days), fulfilled this criterion. In these patients, seven of 21 lesions (33.3%) in four patients were still visible during ongoing ADT (Figs. 8 and 9). A complete imaging response (negative PSMA PET/CT) was observed in two of the six patients with a complete PSA response (patients 4 and 10).

## Discussion

One of the most important clinical questions in PSMA-based imaging concerns the role of ADT. It is known from preclinical research that short-term ADT with LHRH analogues as well as androgen receptor blockers such as bicalutamide and enzalutamide can quantitatively increase PSMA expression in PC cells [11–13]. If this could also be observed in the clinical setting in patients receiving long-term ADT, it would have a substantial positive impact on PSMA imaging as well as radioligand therapy with PSMA ligands. Multivariate analyses including larger patient cohorts with PSMA ligand PET/CT published so far [6, 7] suggest that ^68^Ga-PSMA-11 PET/CT significantly more often shows pathological findings in patients receiving ADT. All these preclinical and clinical reports have tended to convey the impression that ADT increases tracer uptake and thereby leads to improved sensitivity of PSMA-11 PET/CT. This impression is reinforced by the fact that even PSMA ligand PET/CT guidelines suggest that ADT does not need to be paused prior to scanning [17]. However, the results of the current study demonstrate for the first time that long-term and effective ADT significantly decreases tracer uptake on PSMA ligand PET/CT: only 45% of the lesions were still visible during ongoing ADT.

Considering the preclinical data mentioned above, no principal differences were detected between the androgen-deprivation agents administered to our patients and those agents included in the preclinical studies. In some of the studies, the dose of ADT was higher than normal doses administered to patients, though: Evans et al. [11] administered 10 mg/kg of enzalutamide to mice (dose in patients usually 160 mg/day), and Hope et al. [13] administered 10 mg/kg/day of apalutamide to mice (dose in patients 240 mg/day). On the other hand, Hope et al. used 50 mg/day of bicalutamide in the single patient reported, which is in the recommended range for treatment of PC (50–150 mg/day) [13].

The most significant difference between our patients and the studies discussed above is the duration of ADT: while the preclinical studies as well as the single patient of Hope et al. received ADT for 2–30 days [11–13, 18], our patient cohort was treated for an average of 229 days. The shortest duration of ADT in our cohort was 6 weeks (patient 7) which is the closest to the 4 weeks of ADT in the patient of Hope et al. [13]. Although in patient 7 a significantly negative impact of ADT on the results of the PET was shown as well, we strongly believe that the duration of ADT is a key factor in the impact of ADT on PSMA expression. ADT of short duration may increase PSMA expression while long-term ADT probably has the opposite effect. The latter assumption has been observed in a preclinical study by Liu et al. [18]. However, more longitudinal clinical studies are necessary to analyse this issue.

The intensity of tracer uptake in relation to lesion volume was also analysed in this study. This represents a volume-independent estimation of PSMA expression. This analysis frequently demonstrated a decrease in uptake during ongoing ADT, counteracting any remaining assumption that long-term ADT does not negatively affect PSMA expression on PC cells. Overall, we assume that long-term androgen deprivation results in apoptosis of androgen-sensitive PC cells, an effect that was demonstrated by the 1966 Nobel Prize winner Charles Brenton Huggins. In general, the effect of the reduction in tumour volume and the decrease in PSA values caused by apoptosis is accompanied by a negative impact on tumour detection by imaging. The higher probability of a pathological PSMA PET/CT scan in patients receiving ADT found in previous studies was most likely caused by bias in the selection of patients with more advanced tumour stages which would have led to initiation of ADT.

Currently the most accepted indication for PSMA ligand PET/CT is biochemical relapse of PC. Depending on tumour spread, tumour detection can guide locoregional approaches or systemic therapies. In such a setting the highest possible sensitivity is mandatory and physicians as well as patients would rather accept short-term ADT or possibly pausing ADT to optimize the diagnostic accuracy of PSMA ligand PET/CT. Considering the mainly preclinical data discussed above, short-term ADT may eventually increase PSMA expression on PC cells, thereby increasing the sensitivity of PSMA ligand imaging. The findings of all preclinical studies discussed here as well as in the single patient of Hope et al. suggest that there is a functional coupling between PSMA and androgen receptors. However, these studies do not provide any clinical evidence. Therefore, further evaluations are necessary to determine if there is any real clinical coupling between PSMA and androgen receptors. Furthermore, it is well known that LHRH analogues cause an increase in the whole metabolic activity of PC cells. In this context, an increase in uptake of PSMA ligands seems possible and cannot be excluded. However, LHRH analogues as well as androgen receptor blockers such as bicalutamide caused a quantitative increase in PSMA expression in the studies discussed above [11–13].

Previous evaluations have shown that about one third of patients referred for PSMA ligand PET/CT are receiving ADT [6, 7]. We assume that a proportion of them are referred to confirm the presence of recurrent disease. However, according to our results, PSMA PET/CT delineates only a fraction of the tumour disease in patients receiving long-term ADT with a continuous PSA response. Therefore, these scans are not suitable for subsequent therapy planning. Instead, PSMA ligand PET/CT in patients receiving ADT can be used for response assessment. For example, early detection of hormone-resistant lesions can open up new possibilities for individual treatment strategies. This suggestion is potentially supported by our results which showed that in patients with complete PSA remission, 33% of the lesions were still visible (Figs. 8 and 9). This implies that ADT can cause some “cosmetic effects” on PSA levels while viable tumour is still present. It may be that the 12.9% of lesions showing an increase in tracer uptake on PET-2 or those lesions still visible during ongoing ADT despite (complete or incomplete) PSA remission might correlate with those cell clones that become castration-resistant first.

A future strategy could be to perform PSMA ligand PET/CT despite a sufficient PSA response. If the scans show remaining viable (PSMA-positive) tumour lesions, ADT could be augmented by novel androgen signalling inhibitors or taxanes. Otherwise, ADT could be continued without change. Such a strategy would refine and individualize the recommendations derived from the CHAARTED, LATITUDE and STAMPEDE trials, which showed a remarkable survival benefit for primarily metastasized patients by adding therapy during the castration-sensitive stage [19–21]. If the critical cell population, from which future tumour progression is derived, is characterized by continuing PSMA expression during ongoing ADT, PSMA-based radioligand therapy, which has already shown remarkable antitumour activity in the last-line setting [22–26], might also be a powerful therapeutic partner for ADT.

Although controversial from a therapeutic point of view, pausing long-term ADT could help increase the sensitivity of PSMA ligand PET/CT with the aim of visualizing the maximum possible extent of disease. The still-unanswered question is how long does ADT need to be paused to increase the sensitivity of PSMA ligand imaging. To our knowledge, no data are available on how long PC cells need to achieve their natural PSMA status after termination of ADT or whether discontinuing ADT might even cause a temporary PSMA rebound. It is also unclear how PSA status is associated with the degree of PSMA expression. It is known from preclinical studies that PSA and PSMA promoters are regulated in an opposite manner with short-term ADT leading to a decrease in PSA levels and an increase in PSMA expression [12]. On the other hand, it remains a matter of speculation if the full PSMA expression status and therefore the maximum possible tumour visibility on PSMA ligand imaging will be achieved if PSA levels rise to pre-ADT levels.

In six of ten morphologically growing lesions the SUV decreased during ongoing ADT. This observation demonstrates that changes in SUV can occur independently of lesion volume. At least regarding bone metastases, it is known that their volumes can increase after different therapies despite a good PSA response. However, this increase in volume is caused by sclerosis and must not be interpreted as tumour progression. Consequently, the size of bone metastases was excluded from the Response Evaluation Criteria In Solid Tumors (RECIST). According to our experience, we believe that PSMA ligand uptake in tumour lesions represents a more accurate therapy response criterion than lesion size.

The limitations of this study include the low number of patients who could be included in the current analysis despite a large database of more than 1,700 patients, the wide range of ADT duration and the retrospective nature of the evaluation.

Currently, patients with biochemical relapse should be referred for PSMA PET before starting ADT. Future studies are needed to show the impact of short-term ADT in the clinical setting as well as of alterations in PSMA expression after pausing ADT.

### Conclusion

Long-term ADT in patients with castration-sensitive PC demonstrated a significant negative impact on tumour detection on PSMA PET/CT. The majority of PC lesions were no longer visible in patients receiving effective ADT. These findings indicate that, in the setting of biochemical relapse, PSMA PET/CT should preferably be performed prior to starting ADT, with the aim of visualizing the maximum possible extent of disease. PSMA PET/CT in patients receiving ADT can potentially be used for more precise response assessment than can be achieved with PSA alone: early detection of isolated hormone-resistant lesions could change the treatment strategy.
